# Tobacco use and betel quid chewing among adults in Myanmar- estimates and social determinants from demographic and health survey, 2015–16

**DOI:** 10.1186/s12889-021-10347-1

**Published:** 2021-02-03

**Authors:** Chandrashekhar T. Sreeramareddy, Saint Nway Aye, Sunil Pazhayanur Venkateswaran

**Affiliations:** 1 Department of Community Medicine, International Medical University, Bukit Jalil, 57000 Kuala Lumpur, Malaysia; 2 Department of Pathology, International Medical University, Bukit Jalil, 57000 Kuala Lumpur, Malaysia

**Keywords:** Tobacco use, Betel quid chewing, Prevalence, Myanmar

## Abstract

**Background:**

National-level prevalence of tobacco use and betel quid chewing, and associated socio-demographic factors were estimated using first-ever, Myanmar Demographic Health Survey, 2015–16.

**Methods:**

Questions about tobacco smoking, smokeless tobacco use, and betel quid chewing were used to create outcome variables such as tobacco smoking, smokeless tobacco use, and ‘dual use’ (tobacco use and betel quid chewing). Sex-stratified weighted prevalence rates, distribution by socio-demographic factors were presented. Association of demographic factors with tobacco and/or betel quid chewing was assessed by multinomial logistic regression.

**Results:**

Among men, prevalence (%) of tobacco use and betel quid chewing was 40.9 (95% CI 38.1, 42.1) and 58.9 (95% CI 56.3, 61.6) respectively. Among women tobacco use was 3.7 (95% CI 2.0, 4.3) and betel quid chewing 18.2 (95% CI 16.4, 20.0). Among men prevalence of either tobacco or betel quid and ‘dual use’ was 50.4 (95% CI 48.5, 52.3) and 25.0 (95% CI 23.1, 26.8) respectively, whereas among women the corresponding rates were 17.9 (95% CI 16.2, 19.6) and 2.0 (95% CI 1.6, 2.9). Smokeless tobacco use was low (< 5%) in both sexes. Tobacco use and/or betel quid chewing was associated with age, wealth, marital status, and occupation in both sexes. However, the effect sizes were much larger among women for wealth groups. People of older age and lower wealth had a higher odds of being a tobacco user and/or betel quid chewer.

**Conclusions:**

In Myanmar, prevalence of both tobacco use and betel quid chewing was high particularly among men. Tobacco control interventions should be strictly implemented and ‘dual use’ of both tobacco and betel quid should also be urgently addressed.

## Background

Tobacco smoking still stands as the second leading risk factor for disability and death worldwide, despite several evidence-based, anti-smoking interventions [1]. Global prevalence of smoking was estimated at 25% in 2015 with very little decline since 1990 [2, 3]. Smoking prevalence has shown wide sex-wise and geographic differentials and disease burden attributable to smoking is growing in low-income countries [4]. Southeast Asia (SEA) region is home to an estimated 400 million tobacco users causing an estimated 1.2 million smoking attributable deaths annually [4]. Tobacco is also consumed in diverse smokeless forms in South Asia (SA) and SEA [4–6]. Smokeless tobacco (SLT) use is a well-established risk factor for oropharyngeal cancers [7]. In addition, betel quid chewing with or without tobacco leaves along with other varied ingredients is a widely prevalent practice in many parts of Asia including SEA [8]. Betel quid chewing mixed with tobacco greatly increases the risk for bleeding gums, periodontal diseases, oral lesions and oral cancer [8–12].

Tobacco use is common is Myanmar where ‘cheroots’ are also smoked in addition to manufactured cigarettes. Cheroot is a filterless indigenous cigar of Myanmar which has both its ends truncated. Cheroots are hand rolled using bark, stems, roots and sundry leaves. They are filled with a choice blend of tobacco and tied with a red silk thread. Smokeless tobacco use is also very common in Myanmar where eating raw and cured tobacco leaves is most common but tobacco leaves are also mixed with substances such as alcohol, honey, lime etc. [13, 14]. Betel quid chewing is very common in Myanmar as in other SA countries. *Kun-ywet* is the Myanmar term for betel leaf, areca nut is called *Kun-thee*, and the preparation is called *Kun-yar*. Offering and chewing *Kun-yar* or *betel quids* is ceremonial and deeply rooted in the traditional culture of Myanmar, like other countries in SA. Betel quid chewers usually do not add tobacco into the betel quid during the early days. However, at later stages some betel quid chewers mix tobacco leaves into the quid and gradually develop nicotine dependence. In Myanmar, different forms of tobacco flavours, and contents in the betel quid is practiced widely and is believed to be breath-freshening and a mouth-cleansing agent [13, 14]. Research has shown that about 80% of betel quid chewers add tobacco leaves and 9.6% of them had premalignant oral disorders [12].

The Framework Convention on Tobacco Control (FCTC) adopted in 2003 has been ratified by more than 180 countries worldwide [15]. Under the FCTC, monitoring of worldwide use of tobacco by population-based surveys has been prioritized to understand the patterns of tobacco use behaviors, assess the impact of tobacco control measures, and to assist policy changes in tobacco control. Consumption of both smoking and smokeless tobacco products is being recognised as a distinct tobacco use behaviour to combat the tobacco epidemic since it increases the risk of tobacco-induced diseases [16]. Smoking, SLT and betel quid chewing all are known risk factors for oropharyngeal cancers, which may act additively or synergistically [7–10, 17]. In SEA, SLT use is increasing [5, 18] and SLT has been used as a replacement to smoking and tobacco users are also known to switch between these products in an attempt to quit [19, 20]. Studying socio-economic determinants of tobacco use and betel quid chewing also helps to develop targeted interventions towards vulnerable and disadvantaged populations [6]. National-level prevalence of smoking and SLT in SEA are well reported [5, 6] but very few reports are available on betel quid chewing in Myanmar [21, 22]. However, there is a lack of nationally representative surveys for Myanmar reporting on both tobacco use and betel quid chewing. Myanmar participated in its first Demographic and Health Survey (DHS) in 2015–16. We aimed to provide national level estimates for prevalence of tobacco use (smoking and SLT), betel quid chewing and ‘dual use’. We also aimed to assess the socio-demographic determinants of ‘dual use’ (both tobacco use and betel quid chewing).

## Methods

### Data source

The 2015–16 Myanmar Demographic and Health Survey (2015–16 MDHS) was implemented by the Ministry of Health and Sports (MoHS) between December 7, 2015, and July 7, 2016. MDHS was funded by the United States Agency for International Development (USAID) and the Three Millennium Development Goal Fund (3MDG) with technical support from Inner City Fund (ICF International, Inc.) via the DHS Program. The primary objective of the 2015–16 MDHS was to provide up-to-date estimates of basic demographic and health indicators such as fertility, family planning, health, nutrition and so on [23].

### Sampling and sample size

The sampling frame for MDHS consisted of 76,990 primary sampling units (PSUs) spread across the country. A PSU was either a census enumeration area (EA) or a ward or village tract. Geographic locations where internal conflict was ongoing were considered as sensitive areas and were not enumerated during the census. Each PSU had cartographic materials that delineated its geographic location, boundaries, main access points, and landmarks. The sampling frame provided information about administrative subordination by state, region or district, urban or rural, and the estimated number of residential households in each PSU. Institutional populations, such as hotels, barracks, and prisons were not sampled. However, internally-displaced population camps were included [23]. All men and women aged 15–49 years who were permanent residents of the selected households or visitors who stayed in the households the night before the survey date were interviewed. Men were interviewed in only half of the selected households [23].

The final samples of households in MDHS were selected by stratified two-stage random sampling method. Stratification by urban-rural was done and clusters were selected by probability proportionate to size technique. Sampling weights were calculated based on sampling probabilities calculated separately for each sampling stage and for each cluster. The household head provided responses to the general questions about the household and listed all the members of the household. Men and women enlisted were eligible for the interview, if they were either permanent residents or visitors who stayed in that house the night before. Trained interviewers collected information about demographic and socio-economic factors and health status. Questions about tobacco use and betel quid chewing were asked to all eligible men and women [24, 25].

### Outcome variables

The following six questions were asked to gather information about tobacco use and betel quid chewing in Myanmar:

1. Do you currently smoke cigarettes? (response as ‘yes’ or ‘no’)
2. In the last 24 h, how many cigarettes did you smoke? (response as numerical)
3. Do you currently smoke or use any other type of tobacco? (response as ‘yes’ or ‘no’)
4. What (other) type of tobacco do you currently smoke or use? (options provided were pipe, cigar, cheroot, chewing tobacco, snuff, others)
5. Do you currently chew betel nuts? (response as ‘yes’ or ‘no’)
6. In the last 24 h, how many pieces did you chew? (response as numerical)

Each respondent was classified as ‘tobacco smoker’, if the response was ‘yes’ to the first and third questions and they responded as ‘pipe,’ ‘cigar’, or ‘cheroot’ to the fourth question. The respondents were classified as ‘SLT user’ if the response to the fourth question was any form of SLT, including ‘chewing tobacco, ‘and ‘snuff’. If they responded as ‘yes’ to fifth question, classified as ‘betel quid chewer’. To assess the socio-demographic determinants, we created a nominal outcome variable as ‘non-user’, either ‘tobacco user’ (any type of smoking or smokeless tobacco) or ‘betel quid chewer’ and ‘dual users’ (both tobacco use and betel quid chewing).

### Explanatory variables

For multinomial logistic regression analyses, we used age in completed years, marital status, education, occupation, wealth index, and questions on weekly frequency of exposure to mass media (1) reading newspaper or magazine, 2) listening to radio and 3) watching television). Exposure to these media was measured as ‘at least once a week’, ‘less than once a week’ and ‘not at all’ and we scored these responses as 2, 1 and 0 respectively. We generated a score by summing up the responses to the three questions to quantify the frequency of exposure to mass media. The possible scores ranged from 0 to 6. Marital status was classified as ‘never married’, ‘currently married’ and ‘single’. ‘Single’ constituted being separated, divorced, and widowed. Educational level was classified as ‘no education’, ‘primary’, ‘secondary,’ or ‘higher.’ Household wealth index, considered as a reliable proxy for household economic status [26] was calculated based on a standard set of household assets, dwelling characteristics, and ownership of consumer items as observed by the interviewer. Participants were ranked on the basis of their household wealth score by dividing them into quintiles where the first quintile was the poorest 20% of the households and the fifth quintile was the wealthiest 20% [27]. Spatial variables such as urban-rural, and state or region or district were not used in the multinomial logistic regression analyses since the sample selection was stratified by urban-rural and the sample distribution by administrative units (states/regions) was similar.

### Ethics statement

The institutional review boards of ORC Macro International Inc. and participating institutions in Myanmar provided ethical clearance for MDHS. In each survey, participants were informed about voluntary participation and confidentiality of information and they could refrain from responding to any of the questions. Before each interview, details of the survey were explained, and informed consent was obtained. Written consent was not obtained since no intervention was applied to the participants.

### Data analysis

All analyses were stratified by sex. Unweighted and weighted proportions and weighted prevalence rates for types of tobacco smoking, SLT use and betel quid chewing were estimated. Sample weights were applied during estimation to account for the complex sampling design used in MDHS. Weighted prevalence estimates of tobacco smoking, SLT use and betel quid chewing were calculated by the age groups, marital status, occupation, education, and wealth quintiles. Multinomial logistic regression analyses were done to assess socio-demographic factors associated with ‘dual use’, tobacco use, betel quid chewing as compared to non-user (reference category). Using *‘svy’* command on STATA/IC version 11.2 the multinomial logistic regression model we included for age, wealth, marital status, occupation and score for exposure to mass media in the models. However, education was not included in the full model since it was correlated with wealth and occupation. Adjusted odds ratios their 95% confidence intervals, and *p*-values were calculated.

## Results

### Sociodemographic characteristics of survey population

Sociodemographic characteristics presented as both weighted and unweighted numbers and proportions are shown in Table 1. A total of 17,622 subjects were surveyed of whom 27% were men and 73% were women and their mean age was 31.3 years and 31.6 years respectively. More than half of respondents were aged between 20 and 40 years. More than two-thirds of respondents were rural residents. About 80% of men and women were educated up to primary and secondary level. About a third were never married and 61% were currently married. Men and women were nearly equally distributed by household wealth quintiles. Men were mostly working in agricultural and manual work (77.0%) and women were unemployed/domestic workers (28.8%) and manual workers (29.8%).

**Table 1:** Sociodemographic characteristics of survey population

	Men (*N* = 4737)		Women (*N* = 12,885)	
	Unweighted Number (%)	Weighted Number (%)	Unweighted Number (%)	Weighted Number (%)
**Age**	Mean = 31.27 (10.1) Median = 31 (22, 40)		Mean = 31.6 (9.88) Median = 32 (23, 40)	
**Age groups**				
15–20	926 (19.5)	891 (18.8)	2220 (17.3)	2205 (17.1)
21–30	1380 (29.1)	1373 (29.0)	3815 (29.6)	3771 (29.3)
31–40	1315 (27.8)	1351 (28.5)	3843 (29.8)	3929 (30.5)
41–49	1116 (23.6)	1122 (23.7)	3007 (23.3)	2980 (23.1)
**Education**				
No education	559 (11.8)	576 (12.2)	1592 (12.0)	1606 (12.5)
Primary	1630 (34.4)	1684 (35.5)	5129 (40.0)	5305 (41.2)
Secondary	2224 (47.0)	2139 (45.1)	4838 (37.7)	4647 (36.0)
Higher	324 (6.8)	340 (7.2)	1324 (10.3)	1325 (10.3)
**Marital status**				
Never married	1695 (35.9)	1645 (34.8)	4146 (32.3)	4273 (33.3)
Currently married	2916 (61.7)	2955 (62.6)	7870 (61.4)	7751 (60.4)
Divorced/Separated/Widowed	114 (2.4)	125 (2.6)	811 (6.3)	803 (6.3)
**Wealth Index**				
Poorest	904 (19.1)	890 (18.8)	2364 (18.3)	2274 (17.6)
Poorer	933 (19.7)	916 (19.3)	2451 (19.0)	2408 (18.7)
Middle	1016 (21.4)	980 (20.7)	2633 (20.4)	2633 (20.4)
Richer	995 (21.0)	986 (20.8)	2739 (21.3)	2702 (21.0)
Richest	889 (18.8)	965 (20.4)	2698 (21.0)	2868 (22.3)
**Type of residence**				
Rural	3416 (72.1)	3387 (71.5)	9100 (70.6)	9117 (70.8)
Urban	1321 (27.9)	1351 (28.5)	3785 (29.4)	3768 (29.2)
**Occupation**				
Unemployed/domestic work	291 (6.2)	295 (6.2)	3711 (28.8)	3550 (27.6)
Professional/service/sales	787 (16.6)	820 (17.3)	3429 (26.6)	3317 (25.8)
Agricultural work	1256 (26.6)	1274 (27.0)	1880 (14.6)	1847 (14.4)
Manual worker	2394 (50.6)	2340 (49.5)	3835 (29.8)	4141 (32.2)

### Sex-wise distribution of types of tobacco products and betel quid chewing

Unweighted and weighted numbers and their proportions of types of tobacco products consumed for multiple tobacco products are shown in Table 2. More than half of the men (59%) chewed betel quid, about a third (31.7%) of the men smoked cigarettes and 14.4% of men smoked pipe/cigar/cheroot, whereas users of other types of tobacco products was less than 10%. Overall, 40% of men were smoking tobacco while only 2% used smokeless tobacco. Nearly 50% the men either chewed betel quid or consumed tobacco products, while 25% of them consumed both. Only 24.6% of the men were non-users of either tobacco or betel quid. On the other hand, the tobacco use among women was very low (3.7%) whereas betel quid chewing was 18.2%. Compared to men, the proportion of women who smoked was low for cigarettes (1.7%) and cheroots (2.1%) as well as betel quid chewing (18.2%). Overall, 3.7% of women smoked tobacco and only 0.2% used smokeless tobacco. A majority (80.1%) of the women were non-users, about 17.9% either used tobacco or chewed betel quid and only 2.0% were ‘dual users.’

**Table 2:** Weighted and unweighted frequencies and proportions of various behaviors related to tobacco products and betel quid use among men and women in Myanmar, 2015–16

	Men = 4737				Women = 12,598			
	Unweighted		Weighted		Unweighted		Weighted	
	Number	%	number	%	Number	%	Number	%
Cigarette smoking	1594	33.7	1504	31.7	277	2.2	214	1.7
Smoking pipe/cigar/cheroot/others	714	15.0	683	14.4	399	3.1	266	2.1
Chewing Tobacco	63	1.3	57	1.2	22	0.2	22	0.2
Snuff	33	0.7	44	0.94	2	0.02	3	0.02
Betel quid chewing	2779	58.7	2792	59.0	2661	20.7	2343	18.2
Tobacco smoking (1 + 2)	1976	41.7	1899	40.1	653	5.1	467	3.7
Smokeless tobacco use (3 + 4)	91	1.9	95	2.0	24	0.2	25	0.2
Any type of tobacco use (1 + 2 + 3 + 4)	2019	42.6	1940	40.9	674	5.2	491	3.8
Non-use of either tobacco or chew betel quid	1179	24.9	1166	24.6	9887	76.7	10,316	80.1
Use of either tobacco or betel quid	2318	48.9	2410	50.4	2660	20.7	2304	17.9
Use of both tobacco and betel quid	1240	26.2	1161	25.0	336	2.6	263	2.0

### Distribution of weighted prevalence rates of tobacco smoking, SLT use, ‘dual user’ by socio-economic and demographic factors

Distribution of weighted prevalence rates (%) of tobacco use and betel quid chewing by socio-demographic factors are shown in Table 3. Weighted prevalence rates of tobacco use, and betel quid chewing varied by socio-demographic factors among both men and women. There was a clear gradient across education and wealth subgroups. Prevalence of tobacco use was highest among the respondents without education compared to those with higher education (50.8 vs. 30.7); from poorest households compared to the richest (45.7 vs. 36.9). Differentials by wealth for betel quid chewing and ‘dual use’ were similar among both men and women. However, the differentials were much wider among women. Betel quid chewing among men did not show any differentials by educational attainment. Both tobacco use and betel quid chewing rates were higher among divorced, widowed and separated men and women than never married and currently married. An exception was the highest rate of betel quid chewing rates among currently married men. Urban-rural differentials showed an inconsistent pattern among men; however, rural women had a higher prevalence of both tobacco use and betel quid chewing than their urban counterparts. Agricultural and manual workers had a higher prevalence rates of tobacco use as well as betel quid chewing in both sexes.

**Table 3:** Weighted prevalence rates (%) of tobacco use, betel quid chewing and dual use by socio-demographic factors among men and women in Myanmar, 2015–16

	MEN					WOMEN				
	Tobacco use	Betel quid	Non-user	tobacco or betel quid	Dual user	Tobacco use	Betel quid	Non-user	tobacco or betel quid	Dual user
**Overall**	40.9	58.9	24.6	50.4	25.0	3.7	18.2	80.1	17.9	2.04
**Age groups**										
15–20	24.4	41.4	50.3	33.6	16.1	0.5	5.7	93.9	5.9	0.2
21–30	42.8	64.2	20.7	51.6	27.7	1.7	13.0	86.2	13.0	0.9
31–40	44.7	62.8	19.6	53.3	27.1	4.3	23.1	75.1	22.3	2.6
41–49	47.2	61.8	15.1	60.8	24.1	8.2	27.6	68.7	27.2	4.2
**Education×**										
No education	50.8	50.8	21.8	53.2	25.1	9.8	28.1	66.6	28.8	4.6
Primary	41.5	66.3	17.1	55.7	27.2	4.9	24.7	73.1	23.9	3.0
Secondary	37.6	56.4	29.5	46.1	24.3	1.0	11.2	88.4	10.9	0.6
Higher	30.7	51.6	38.5	45.9	18.2	0.4	4.6	95.0	5.0	–
**Marital status**										
Never married	31.5	50.0	37.7	42.3	20.0	0.9	7.3	92.0	7.5	0.4
Currently married	43.6	64.0	17.7	55.3	27.0	4.8	22.8	74.8	22.4	2.7
Divorced/Separated/Widowed	69.5	55.5	16.0	40.9	43.0	6.2	30.9	67.0	28.7	4.2
**Wealth Index**										
Poorest	45.7	64.8	16.8	54.0	29.1	10.2	35.0	60.6	33.6	5.8
Poorer	41.0	62.1	21.3	51.7	26.9	5.0	22.8	75.0	22.3	2.7
Middle	39.3	59.5	24.4	51.3	24.2	2.9	16.2	82.3	16.3	1.4
Richer	38.0	56.1	27.9	49.1	23.0	1.5	12.4	86.7	12.6	0.7
Richest	36.9	52.9	31.7	46.1	22.0	0.6	8.2	91.5	8.2	0.3
**Type of residence**										
Rural	39.4	59.7	23.7	51.9	24.4	46.8	20.4	77.4	19.8	2.7
Urban	41.7	57.1	27.0	46.6	26.4	1.1	12.8	86.4	13.2	0.4
**Occupation**										
Unemployed/domestic work	24.4	29.8	59.8	25.6	14.5	3.5	18.9	79.7	17.8	2.3
Professional/service/sales	39.3	57.3	27.0	48.9	24.1	2.1	16.9	82.0	16.7	1.3
Agricultural work	38.6	60.3	23.6	50.8	25.6	4.6	22.9	74.6	23.2	0.22
Manual worker	43.0	62.3	20.0	53.9	26.1	4.5	16.6	81.1	16.5	2.4

Chi square test was used to test the statistical significance of differences in tobacco use, betel quid chewing and dual use by sociodemographic factors all factors were statistically significant at p < 0.001 or *p* < 0.01 (betel quid by type of residence) except for tobacco use type of residence (*p* > 0.05)

### Association of tobacco use and betel quid with socio-demographic factors

Association of being both a tobacco user as well as a betel quid chewer (‘dual use’) and use of one product only as compared to non-use with socio-demographic factors was tested using multinomial logistic regression analyses and the results are shown in Table 4. In both sexes, older age was associated with being “dual users’ as well use one product only. The odds being tobacco user and/or betel quid chewer was higher in older age (adj. OR 1.0 to 1.1, *p* < 0.001). However, frequency of exposure to media was not associated with either ‘dual users’ or use of one product only (except for tobacco use among men).

**Table 4:** Factors associated with tobacco use and betel quid chewing by multinomial regression analyses

	MEN						WOMEN					
	Betel quid chewer versus non-user		Tobacco user’ versus non-user		dual user’ versus non-user		Betel quid chewer versus non-user		Tobacco user’ versus non-user		dual user’ versus non-user	
	Adj. Odds Ratio (95% CI)	p-value	Adj. Odds Ratio (95% CI)	p-value	Adj. Odds Ratio (95% CI)	p-value	Adj. Odds Ratio (95% CI)	p-value	Adj. Odds Ratio (95% CI)	p-value	Adj. Odds Ratio (95% CI)	p-value
**Age**	1.0 (1.0, 1.1)	< 0.001	1.1 (1.0, 1.1)	< 0.001	1.0 (1.0, 1.1)	< 0.001	1.1 (1.0, 1.1)	< 0.001	1.1 (1.1, 1.2)	< 0.001	1.1 (1.1, 1.2)	< 0.001
**Marital status**												
Divorced/separated/ widowed	1		1		1		1		1		1	
Currently married	1.5 (0.7, 3.4)	0.271	0.4 (0.2, 0.9)	0.028	0.3 (0.2, 0.7)	0.002	0.4 (0.3, 0.5)	< 0.001	1.0 (0.5, 2.2)	0.986	0.3 (0.2, 0.7)	0.001
Never married	2.1 (1.0, 4.5)	0.049	0.5 (0.3, 1.2)	0.111	0.5 (0.3, 1.1)	0.089	0.8 (0.6, 0.9)	0.016	1.4 (0.8, 2.6)	0.272	0.8 (0.5, 1.2)	0.227
**Wealth Index**												
Richest	1		1		1		1		1		1	
Richer	1.2 (0.9, 1.7)	0.291	1.2 (0.8, 1.7)	0.448	1.2 (0.8, 1.72)	0.403	1.7 (1.3, 2.2)	< 0.001	3.5 (1.7, 7.3)	0.001	2.4 (1.1, 5.4)	0.036
Middle	1.4 (0.9, 2.0)	0.081	1.3 (0.8, 1.9)	0.259	1.4 (0.9, 2.1)	0.124	2.4 (1.8, 3.0)	< 0.001	6.2 (2.9, 13.4)	< 0.001	5.0 (2.3, 10.9)	< 0.001
Poorer	1.6 (1.1, 2.3)	0.025	1.4 (0.9, 2.1)	0.141	1.6 (1.1, 2.5)	0.020	3.6 (2.8, 4.6)	< 0.001	11.3 (5.4, 23.8)	< 0.001	11.6 (5.3, 25.3)	< 0.001
Poorest	1.9 (1.2, 3.0)	0.005	1.8 (1.2, 2.9)	0.007	2.2 (1.4, 3.5)	0.001	7.3 (5.5, 9.6)	< 0.001	32.4 (15.3, 68.6)	< 0.001	34.4 (15.9, 74.6)	< 0.001
**Occupation**												
Manual worker	1		1		1		1		1		1	
Professional/service/ sales	0.3 (0.2,0.4)	< 0.001	0.4 (0.3, 0.7)	0.001	0.3 (0.2,0.6)	0.000	1.5 (1.2, 1.9)	< 0.001	0.9 (0.6, 1.3)	0.587	1.5 (1.0, 2.1)	0.037
Agricultural work	0.8 (0.6, 1.1)	0.108	0.7 (0.5, 1.1)	0.123	0.8 (0.6, 1.1)	0.210	1.7 (1.3, 2.1)	< 0.001	0.9 (0.6, 1.6)	0.828	1.1 (0.7, 1.8)	0.662
Unemployed/ domestic work	0.8 (0.6, 1.0)	0.065	0.7 (0.5, 0.9)	0.019	0.7 (0.5, 1.0)	0.045	1.5 (1.1,1.92)	0.008	1.1 (0.7, 1.7)	0.794	0.9 (0.6, 1.5)	0.782
**Exposure to media**	1.0 (0.9, 1.2)	0.960	0.8 (0.7, 0.9)	0.017	1.0 (0.9, 1.2)	0.858	1.0 (0.9, 1.1)	0.450	1.0 (0.8, 1.1)	0.642	0.9 (0.8, 1.1)	0.274

‘Dual user’- tobacco user as well as betel quid chewer

Among men being a tobacco user and/or betel quid chewer was associated with wealth index, marital status, and occupation. Men who were currently married had a lower odds of being ‘dual users’ (adj. OR 0.3 (95% CI 0.2, 0.7) as compared to divorced/separated/widowed. Men who belonged to the occupational group professional/service/sales had a lower odds of being ‘dual users’ (adj. OR 0.3 95% CI 0.2, 0.6) as compared to manual workers. Men from poor and poorest households had higher odds of being users of one product only as well as ‘dual users’ (adj. OR 1.4 to 2.2).

Among women being users of one product only and ‘dual use’ was associated with marital status, wealth index and occupation. Being currently married was protective for being tobacco user and/or betel quid chewer. Women currently married had a lower odds of being betel quid chewer (adj. OR 0.42, 95% CI 0.3, 0.5) and ‘dual users’ (adj. OR 0.3, 95% CI 0.2, 0.7). As compared to women who were manual workers all other occupations had a higher odds of being betel quid chewer (adj. OR 1.5 to 1.7) whereas women in the occupational group professional/service/sales had 1.5 times higher odds of being ‘dual users.’ Wealth index was strongly associated with being tobacco user and/or betel quid chewer with higher effect size than that for men. As compared to women from richest households, women in richer to poorest households had 1.7–7.3 times higher odds of being users of being betel quid chewer; 3.5–32.4 times higher odds of being tobacco user. Likewise compared to women from richest households, women in richer to poorest households had 2.4–34.4 times higher odds of being ‘dual users.’ There was a clear gradient across the wealth groups the odds of being tobacco user and/or betel quid chewer increased from the richest to the poorest.

## Discussion

Overall national-level estimates of tobacco use, betel quid chewing, and dual usage is a first comprehensive report for Myanmar. We found that prevalence of betel quid chewing was higher than tobacco use in both sexes. Tobacco use (41.7% vs. 5.1%) and betel quid chewing (58.7% vs. 20.7%) was higher among men than women. SLT use was very low; hence, most tobacco users smoked cigarettes and/or cheroots. Nearly half the men and fifth of women either used tobacco products or chewed betel quid. Notably, a quarter of the men were ‘dual users.’ ‘Dual use’ was associated with increasing age, lower wealth and lower education in both sexes; the effect sizes of these associations were higher among women than men indicating that wider socio-economic differentials in tobacco use and betel quid chewing exist among women.

This national report is helpful for monitoring of the tobacco use pattern over the coming years and for comparisons with previous sub national studies in Myanmar and those with neighbouring countries. Prevalence of tobacco smoking and betel quid chewing among men and women in Myanmar are reported in WHO-STEPS survey (2009) [28] and National Survey on Diabetes Mellitus and Risk Factors for Non communicable Disease (2014) [29]. The reported prevalence rates in these surveys are comparable to Myanmar DHS estimates. Other survey reports available for Myanmar are Sentinel Prevalence Studies of Tobacco in years 2001, 2004 & 2007 which showed that prevalence of tobacco smoking was decreasing from years 2001 through 2007 but smokeless tobacco use (inclusive of betel quid) increased during the same time period [30]. Betel quid use rates among men and women in Myanmar DHS was higher than Nepalese men (58.7% vs. 43.6%) and Indonesian women (20.7% vs. 46.8%) reported in Asian Betel-Quid Consortium Study 2009–10 [8]. Previous reports from SA and SEA [6, 16] have shown that smoking, tobacco use and dual tobacco use varied among the countries; smoking and smokeless tobacco use rates in Myanmar are comparable to India, Pakistan and Nepal [6, 16]. Overall, tobacco smoking rates among men are comparable to neighbouring countries [31] but rates among women are increasing; yet the disparity between men and women was as high as in other SA countries [32]. Prevalence of ‘dual use’ among Myanmar men is much higher than men from other Asian countries [16]. Reports from other Asian countries, do not include betel quid to define ‘dual use’. In Myanmar, being an older adult, poor, less educated, currently married and manual workers had higher odds of ‘dual use’ as in a previous studies [6, 16].

Myanmar tobacco control program framed in 2000 focussed on smoking tobacco products only. Tobacco control was initiated in 2004 when Myanmar ratified the WHO-FCTC [33] and Myanmar Tobacco Control Law of 2006 covers all forms of tobacco consumption [34]; yet tobacco control in Myanmar is still in very early stages [35]. Considering an overall prevalence which has even shown an increase [31] and widespread prevalence of betel quid chewing [13], Myanmar is unlikely to achieve its Global Action Plan’ target 5 of 30% relative reduction of current tobacco use among adults aged 15 years and above by 2025 [36]. Myanmar faces an unprecedented challenge of ‘dual use’ of tobacco use (smoking and smokeless tobacco) and betel quid chewing. Antismoking measures such as smoking ban in public places and prohibition of tobacco product advertising and promotion, health warnings on tobacco packages were implemented after a long delay of WHO-FCTC ratification and passing of tobacco control law [37, 38]. In our analyses mass media exposure was not associated with tobacco use or betel quid chewing possibly the associated was masked by wealth and education strongly associated with tobacco use. Betel quid (areca nut, catechu, slaked lime often mixed with tobacco leaves) is a deeply rooted in the culture and tradition of Myanmar is often consumed during ceremonial occasions such as weddings and offered to guests visiting homes. Myanmar people believe that betel quid is not as harmful as smoking and chewing raw tobacco among rural folks is common who believe that tobacco leaves are breath-freshening and mouth-cleansing [14]. Low cost, manufactured, sales, and marketing mainly by the unorganised sector makes smokeless tobacco more challenging to regulate [13].

This is a first comprehensive report on a nationally representative sample providing the most current estimates of tobacco use and betel quid chewing in Myanmar. National surveys done in Myanmar [29, 31, 35], did not provide detailed analyses of distribution and social determinants of tobacco smoking, SLT use and betel quid chewing. Tobacco use prevalence estimates by sex are comparable with other DHS-based estimates from the region [6, 16] and our estimates will serve as a bench mark for monitoring the progress towards Global non-communicable disease prevention target achievement [36] and for the global tobacco surveillance system [39].

Our results should be interpreted in the light of some limitations of DHS data arising from the survey design and questionnaire contents. Firstly, our prevalence among adults aged 15–49 would be an underestimate, if those aged > 49 years had higher rates of tobacco use. Secondly, the DHS questionnaire had limited questions covering current use only; this did not allow us to estimate the detailed indicators such as former use, age at initiation, and quit attempts. Thirdly, studies about SLT use in Myanmar have reported about several chewable products which may or may not have been included betel quid chewing [5, 14]. However, Myanmar DHS asked a separate question about betel quid chewing without details about mixing betel quid with tobacco; hence, we could not report betel quid chewing as SLT, instead we reported it as a separate behaviour. A recent study has reported that about 84% of the betel quid chewers used tobacco along with it [40]. As a result, our estimates on SLT were likely underestimated because DHS survey questions did not ask about the tobacco leaves consumed along with betel quid. Fourthly, DHS being a cross-sectional in design, temporality of reported associations cannot be inferred. Fifthly, association of ‘dual use’ versus either use and non-use with occupation was in opposite directions between men and women. Possible reasons for this could be misclassification of occupational groups and/or a residual confounding. Lastly, tobacco use being a self-report, we cannot rule out its under-reporting in conservative societies of Asia. DHS did not verify self-reported tobacco use by measuring urinary cotinine levels.

Sequential national-level surveys are needed to monitor socio-economic equalities in tobacco use and betel quid chewing and assist policies and control programs targeting vulnerable groups. Survey questionnaires should include items to identify both current and former users, cessation behaviors and inquire about mixing tobacco leaves into the betel quid which is a common practice in Myanmar. A high proportion of ‘dual users’ of tobacco and betel quid warrants an urgent need for health screening and provide cessation counselling especially for ‘dual users.’

## Conclusions

Tobacco use prevalence rate is alarmingly high among Myanmar men. Betel quid chewing rate is also very high among men and to a lesser extent among women. Stricter implementation and effective enforcement of existing tobacco control measures is necessary particularly among the least educated, and poorer households. Regular Surveys covering tobacco use and MPOWER indicators are needed to monitor tobacco use and betel quid chewing and the implementation of tobacco control strategies.

## Data Availability

The data of Myanmar Demographic and Health Survey are available from measuredhs website and were provided to the authors up on a written request. The files used for analyses and writing this manuscript are available from the authors up on request.

## Abbreviations

DHS: Demographic and Health Survey
SA: South Asia
SEA: South-east Asia
SLT: Smokeless tobacco
FCTC: Framework Convention on Tobacco Control
MoHS: Ministry of Health and Sports
3MDG: Three Millennium Development Goals
ICF: Inner City Fund
PSU: Primary Sampling Unit
EA: Enumeration Areas
ORC: Opinion Research Corporation
OR: Odds Ratio
MPOWER: Monitoring, Protection, Offering, Warning, Enforcing, Raising
WHO: World Health Organisation
STEPS: STEP-wise approach to surveillance
